# Performance of dispersion-corrected density functional theory for thermochemistry and non-covalent interactions

**DOI:** 10.1186/1758-2946-4-S1-P56

**Published:** 2012-05-01

**Authors:** Waldemar Hujo, Stefan Grimme

**Affiliations:** 1 Mulliken Center for Theoretical Chemistry, Institute for Physical and Theoretical Chemistry, University of Bonn, Beringstr. 4, D-53115 Bonn, Germany

## 

The accuracy of non-local van der Waals density functional [1] is tested for the thermochemical properties of 1200+ atoms and molecules in the GMTKN30 database. Five (hybrid)GGA functionals are augmented by the non-local (NL) part of the VV10 functional. The widely used atom-pair wise dispersion correction DFT-D3 [2] is considered for comparison. The addition of the NL dispersion energy definitely improves the results of all tested short-range functionals. Based on little empiricism and basic physical insight, DFT-NL can be recommended as robust electronic structure method.

For more detailed insight into non-covalent bonding, potential energy curves [3] for five complexes with weak to medium strong hydrogen bonds have been computed with dispersion corrected DFT methods VV10, DFT-D3 and vdW-DF2 [4]. All dispersion corrected methods perform reasonably well for these hydrogen bonds. For the fluorinated complexes, the VV10 method gives remarkably good results. The vdW-DF2 method yields good interaction energies similar to the other methods, but fails to provide accurate equilibrium separations. For large-scale applications we can recommend DFT-D3 based structure optimizations with subsequent checking of interaction energies by single-point VV10 computations.
